# Phenotyping of Mitral Valve Prolapse Without Severe Mitral Regurgitation Using Electrocardiographic and Echocardiographic Data

**DOI:** 10.1016/j.jacep.2025.06.001

**Published:** 2025-07-16

**Authors:** Lionel Tastet, Minhaj U. Ansari, Joshua P. Barrios, Luca Cristin, Rohit Jhawar, Amy Rich, Dwight Bibby, Qizhi Fang, Farzin Arya, Geoffrey H. Tison, Francesca N. Delling

**Affiliations:** Department of Medicine (Cardiovascular Division), University of California-San Francisco, California, USA.

**Keywords:** arrhythmia, clinical outcomes, hierarchical clustering, machine learning, mitral valve prolapse

## Abstract

**BACKGROUND:**

Arrhythmic risk stratification in mitral valve prolapse (MVP) without significant mitral regurgitation (MR) remains elusive. Unsupervised machine learning may reveal phenotypic variation among arrhythmic MVP without severe MR.

**OBJECTIVES:**

In this study, the authors hypothesized that hierarchical clustering of echocardiographic and 12-lead electrocardiographic (ECG) parameters alone could identify MVP phenotypes without severe MR associated with sustained ventricular arrhythmia and excess mortality.

**METHODS:**

The authors identified 343 consecutive MVPs (58 ± 16 years; 51% female) with ≤moderate MR and comprehensive echocardiographic, 12-lead and ambulatory ECG data. They used hierarchical clustering analysis to identify distinctive MVP phenotypes and investigated their association with: 1) arrhythmic events (sudden cardiac arrest, ventricular fibrillation/tachycardia, or frequent ventricular ectopy); and 2) overall mortality (mean follow-up: 5.4 ± 2.7 years).

**RESULTS:**

Three clusters were identified: Cluster 1 (83% of MVP cases), Cluster 2 (9%), and Cluster 3 (8%). Despite mostly trace/mild MR, Cluster 3 exhibited more abnormal parameters of left atrial (LA) and left ventricular structure and function compared with Clusters 1 and 2 (all *P* < 0.001). Top clustering features included ECG intervals, LA systolic strain, and LA function index. Arrhythmic presentations (n = 77) were identified in 19%, 38%, and 43% of Clusters 1, 2, and 3 (*P* < 0.001), respectively. Compared with Cluster 1, Clusters 2 (HR: 5.01; *P* < 0.001) and 3 (HR: 5.85; *P* < 0.001) had significantly increased mortality risk.

**CONCLUSIONS:**

Hierarchical clustering based on standard ECG and echocardiographic data alone identifies 3 MVP clusters with distinct arrhythmic profiles and excess mortality, highlighting LA function as a novel risk parameter in MVP without significant MR.

## CENTRAL ILLUSTRATION

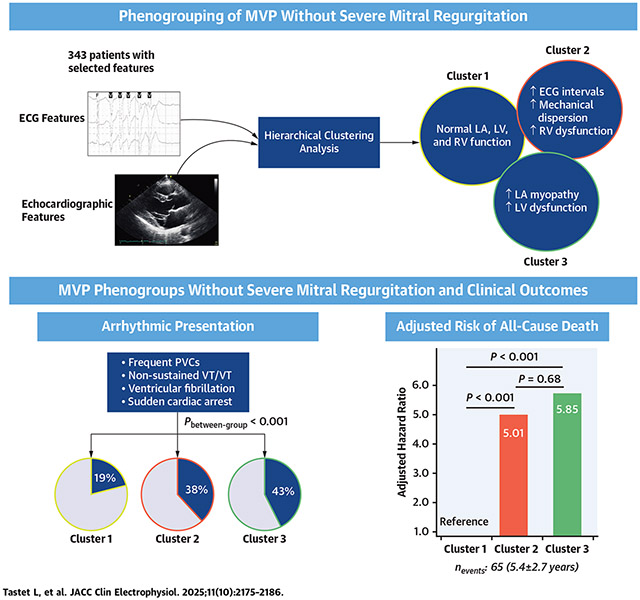

AI-Integrative Approach for Phenogrouping and Risk Stratification of MVP

(Top) Unsupervised ML approach with a hierarchical clustering algorithm applied to ECG and echocardiographic features was used to identify distinct clusters of MVP. (Bottom) Clinical validation of the hierarchical clustering algorithm to identify variation in arrhythmic presentation between clusters and to predict risk of mortality excess during follow-up. ECG = electrocardiography; LA = left atrial; LV = left ventricular; LVEF = left ventricular ejection fraction; LV-GLS = left ventricular–global longitudinal strain; LV-MD = left ventricular mechanical dispersion; ML = machine learning; MVP = mitral valve prolapse; PASP = pulmonary artery systolic pressure; PVC = premature ventricular contraction; RV = right ventricular; VT = ventricular tachycardia.

Mitral valve prolapse (MVP) is a common valvulopathy affecting 2% to 3% of the general population.^1,2^ MVP is generally considered benign in the absence of severe mitral regurgitation (MR).^3-5^ However, a non-negligible subset of individuals with MVP (0.14%-1.8% yearly) are at risk of ventricular arrhythmic complications, sudden cardiac arrest (SCA), and excess mortality, including sudden cardiac death (SCD).^1,6-8^ Several previous studies have reported that less than one-third of MVP-related SCD/sustained ventricular arrhythmia cases are linked to “traditional” risk factors including severe MR.^1,9-12^ Moreover, replacement fibrosis related to abnormal valvular-myocardial mechanics, mitral annular disjunction (MAD) and traction by the prolapsing leaflets, is identified only in 30% of autopsy-defined SCD cases in MVP,^13^ underscoring the need for alternative parameters able to identify the majority of high-risk individuals.

Recently, clustering algorithms applied to echocardiographic and cardiac magnetic resonance (CMR) parameters showed promise in identifying distinct MVP phenotypes associated with replacement myocardial fibrosis, as detected using late gadolinium enhancement, and cardiovascular events. ^14,15^ However, these studies either included MVPs with moderate-to-severe MR or selected MVPs undergoing CMR.^14,15^ Hence, the generalizability of these algorithms to unselected MVPs without significant MR is unclear. Furthermore, electrocardiographic (ECG) repolarization abnormalities have been described in arrhythmic mitral valve prolapse (AMVP), although they are not a consistent finding.^16^ Given the complex and heterogeneous nature of AMVP, the combination of ECG and echocardiographic features beyond left ventricular (LV) assessment and inclusive of left atrial (LA) and right ventricular (RV) structure and function could provide a comprehensive approach uncovering natural phenotypic variations within AMVP. In this context, hierarchical clustering offers a relatively straightforward tool to implement, interpret, and reveal distinct patterns within datasets, contrasting with complex learning models.^16^

We hypothesized that 1) unsupervised clustering algorithms can identify different clusters of MVP without severe MR based on ECG/echocardiography alone and independently of CMR data; and 2) identified clusters are associated with distinct arrhythmic risk and excess mortality profiles.

## METHODS

### STUDY POPULATION.

**FIGURE 1:**
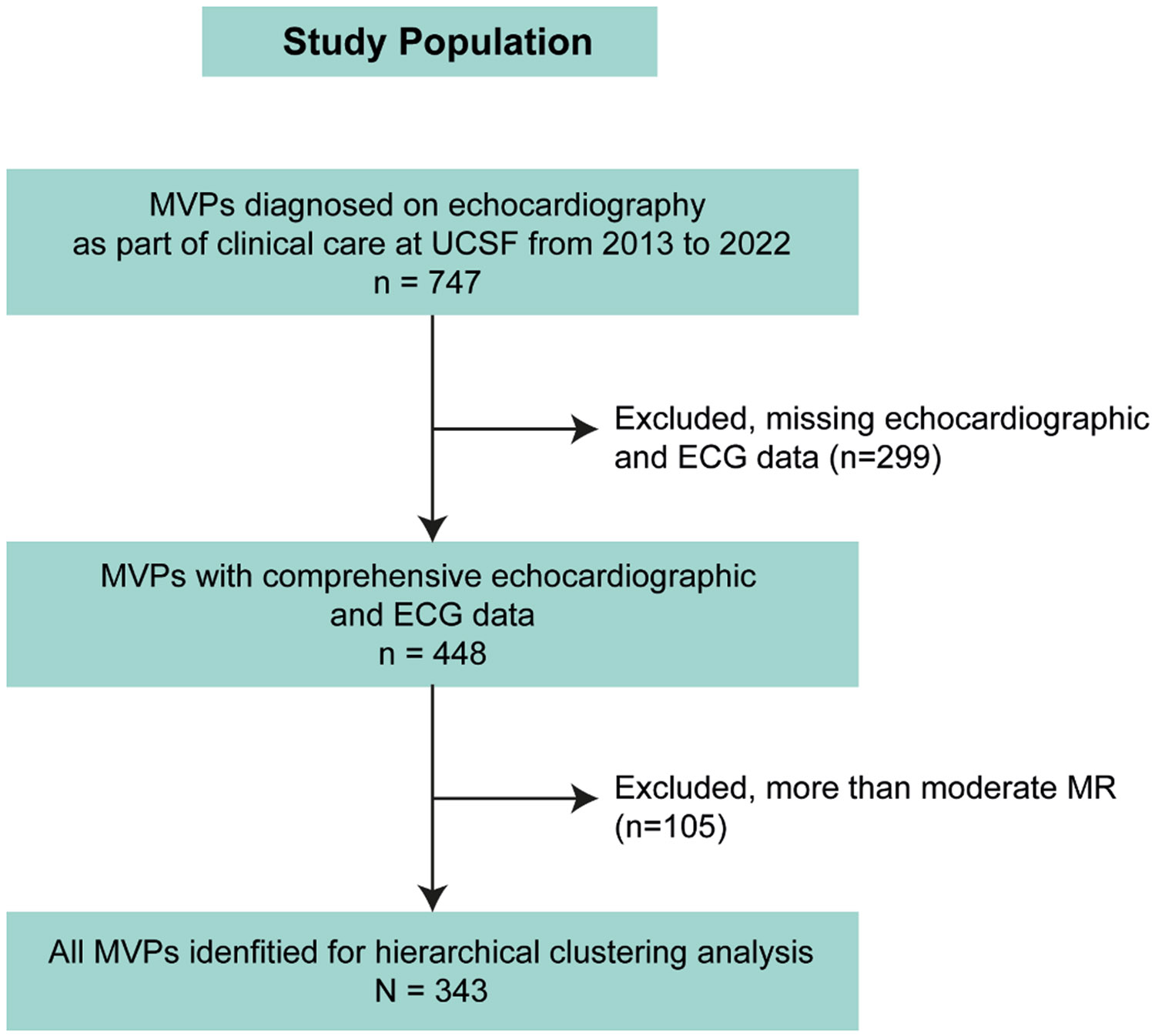
Study Design Study flow chart and design for HCA using unsupervised ML. ECG = electrocardiography; HCA = Hierarchical Clustering Analysis; ML = machine learning; MR = mitral regurgitation; MVP = mitral valve prolapse; UCSF = University of California-San Francisco.

We identified 343 individuals with MVP as part of clinical care at the University of California-San Francisco (UCSF) from 2013 to 2022. Patients were included if they had comprehensive clinical, 12-lead ECG, ambulatory ECG (48-hour Holter or 2-week event monitor), and transthoracic echocardiographic data (Figure 1). Patients with missing echocardiographic or ECG data were excluded. Patients were further excluded if they had a previous mitral valve (MV) intervention, mitral regurgitation (MR) grade >moderate, aortic valve disease (defined as the presence of moderate or greater aortic valve stenosis [ie, aortic valve area ≤1.5 cm^2^ or mean gradient ≥20 mm Hg], aortic valve regurgitation >mild), or a history of ischemic heart disease/myocardial infarction. The UCSF Institutional Review Board approved the study and waived informed consent.

### ARRHYTHMIC EVENTS AND MORTALITY.

Arrhythmic events related to MVP included the composite of: 1) frequent premature ventricular contractions (≤5%) (isolated and/or in couplets/triplets); 2) complex ventricular ectopy including sustained or nonsustained ventricular tachycardia (VT), or ventricular fibrillation; or 3) SCA.^8,16^

All-cause incident deaths were documented through comprehensive medical records review. In addition, incident cases were further ascertained using the National Death Index until 2020. Prevalent deaths occurring before the study period inclusion were excluded.

### ECG DATA.

Standard and interval ECG measurements from the GE MUSE system, as well as raw ECG voltage data, were collected and extracted as previously described.^16^

### ECHOCARDIOGRAPHY.

Two-dimensional transthoracic echocardiograms were performed using commercially available ultrasound systems. All echocardiograms were reviewed by experienced research sonographers (D.B. and F.A.) and a physician with expertise in MVP (F.N.D.). MVP was defined as superior displacement of 1 or both mitral leaflets >2 mm beyond the mitral annulus in a parasternal or apical 3-chamber long-axis echocardiographic view.^1,17^ MVP was classified based on bileaflet or monoleaflet involvement, and MR severity was assessed using a multiparametric approach as recommended by guidelines,^18^ and with grading as follows: no/trace (grade 1), mild (grade 2), moderate (grade 3), or severe (grade 4). MAD was defined as a separation between the basal inferolateral LV myocardium and the LA/mitral leaflet junction, and was assessed in parasternal long-axis view as previously described.^8^ LV and LA dimensions and volumes, LV ejection fraction, pulmonary artery systolic pressure, and RV systolic function (semiquantitative assessment) were measured according to guidelines.^19^ LA volumes were used to calculate the LA emptying fraction and function index.^20^

Speckle-tracking echocardiography (STE) analysis was performed offline using a commercially available software (EchoPAC, GE Healthcare) to assess LA “reservoir” strain as a measure of LA systolic function and left ventricular–global longitudinal strain (LV-GLS) as a measure of LV systolic function.^20,21^ LA strain and LV-GLS were measured as the difference in strain value between ventricular end-diastole (ie, 0 reference point) and ventricular end-systole, resulting in a positive value for LA strain and a negative value for LV-GLS. Mechanical dispersion, a marker of heterogenous LV contraction and electrical dispersion, was calculated as the SD of the time-to-peak strain in 16 LV segments.^22,23^ All patients were in normal sinus rhythm when STE measurements were performed. Variability analyses were previously reported for LA and LV measurements at our institution.^20,24^

### HIERARCHICAL CLUSTERING ANALYSIS.

**FIGURE 2:**
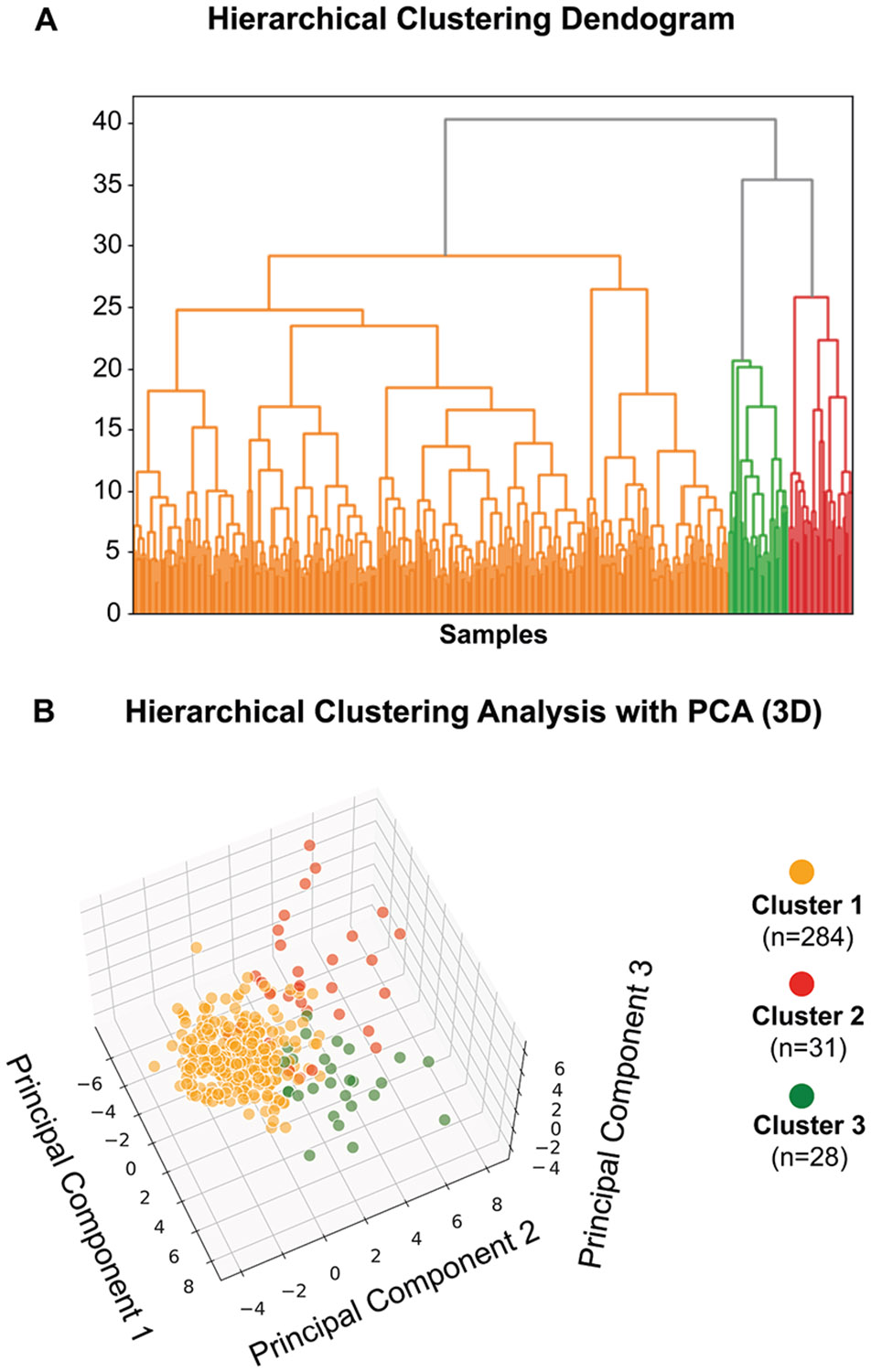
Visualization of HCA Three-dimension distribution between clusters (A). The top 15 of the most important features for the HCA (B). LA = left atrial; RV = right ventricular; other abbreviations as in Figure 1.

Hierarchical Cluster Analysis (HCA) is an unsupervised machine learning (ML) technique used to group similar observations into hierarchical clusters based on a similarity criterion. It starts by treating each data point as its own cluster and iteratively merges the most similar clusters until all data points are merged into a single cluster or a predefined stopping criterion is met. In this study, HCA was used to identify distinct clusters of MVP patients with respect to measurements routinely obtained from echocardiograms and 12-lead ECGs. Continuous parameters were standardized to have mean 0 and SD of 1. Given its clinical relevance as a determinant of AMVP, sex was included as a binary variable but could not be standardized due to its categorical nature. The Ward linkage method,^16^ an agglomerative clustering approach, was used. This method starts with each observation as its own cluster and iteratively merges clusters to minimize overall variance. Three clusters were chosen to test the hypothesis that patients could be separated into low-, medium-, and high-risk groups. The number of clusters was validated by visual inspection of the dendrogram. The value of the y-axis at which clusters merge reflects the increase in variance. The clustering threshold was set at y =32, which resulted in 3 clusters (Figure 2A). Furthermore, principal component analysis was used to visualize the 3 clusters.^25^

To assess the robustness and stability of our clustering results, a sensitivity analysis was performed excluding age and sex from the list of features (Supplemental Methods).

To identify the variables most relevant for cluster identity, a random forest model was fit on the dataset using the same variables to classify patients into the previously identified clusters.^26^ The random forest model assesses feature importance by evaluating how effectively each feature aids in correctly classifying patients across multiple decision trees. The feature importance values from the random forest algorithm were then used as a proxy for the important features of the clustering algorithm.^14^ Clustering and random forest algorithms were implemented using the SciPy libraries^27^ and scikit-learn in Python, with default hyperparameters.^28^

### STATISTICAL ANALYSIS.

Continuous variables were expressed as mean ± SD or median (IQR), and were tested for normality of distribution and homogeneity of variances with the Shapiro-Wilk and Levene tests, respectively. Continuous variables were compared between groups using analysis of variance or the Kruskal-Wallis test, followed by Tukey or Dunn post hoc test, as appropriate. Categorical variables were presented as frequencies and percentages and were compared with chi-square test or Fisher exact test as appropriate.

The estimates of cumulative incidence of all-cause death according to clusters were calculated using the Kaplan-Meier method and compared using the log-rank test. The association between clusters and the risk of all-cause death was determined using Cox proportional hazards models. The multivariable model was adjusted for MV intervention (as a time-dependent variable). Results were presented as HR with 95% CIs. Additional details for clustering sensitivity analyses are reported in the Supplemental Methods. A 2-tailed *P* value <0.05 was considered significant. Statistical analyses were performed with Stata software version 18.0 (StataCorp).

## RESULTS

### STUDY SAMPLE.

From January 2013 to December 2022, a total of 747 individuals from the UCSF MVP Registry were identified (Figure 1). After excluding MVP cases with missing echocardiographic or ECG data (n = 299) and those with more than moderate MR (n = 105), a total of 343 MVPs had complete and already extracted data for exploratory analysis using the unsupervised ML approach (Figure 1). The mean age was 58 ± 16 years and 51% were female. In addition, 85% of MVPs had no or mild MR, 50% bileaflet involvement, and 35% concomitant MAD.

### DEVELOPMENT OF CLUSTERS.

Table 1 shows the list of the 32 features used in our HCA and inclusive of demographic information, 12-lead ECG data, and standard echocardiographic and STE parameters. These variables were selected due to their wide availability in clinical practice or their known prognostic value.^10,24,29,30^ Additionally, among demographic variables, age and sex were included in the HCA given prior studies suggesting age- and sex-related differences in AMVP, particularly a predominance of young female patients among SCD victims.^9,10,31,32^

### DISTRIBUTION AND CHARACTERISTICS OF IDENTIFIED CLUSTERS.

The optimal number of clusters was determined by visually inspecting the dendrogram generated using the hierarchical clustering algorithm (Figure 2A). Hence, the clustering analysis based on an unsupervised ML method revealed 3 distinct clusters of MVP without severe MR (Figure 2A). Cluster 1 included the vast majority of MVP cases (n = 284; 83%), followed by Cluster 2 (n = 31; 9%) and Cluster 3 (n = 28; 8%; Figure 2B).

Table 2 shows the demographic, ECG, and echocardiographic characteristics of the 3 MVP clusters. Although there were no significant differences in ethnicity or cardiovascular risk factors, female sex was more prevalent in Cluster 1 (Table 2). Cluster 2 demonstrated greater QRS and QTc duration on ECG, while the prevalence of T-wave inversion was similar between clusters. No significant differences were observed in MVP subtype (bileaflet vs monoleaflet) or proportion of MAD between clusters. Despite the overall greater proportion of MVPs with trace or mild MR, Cluster 3 showed more pronounced LA and LV remodeling and dysfunction, with larger LA volume index (*P* = 0.002), lower LA reservoir strain (*P* < 0.001), reduced LV-GLS (*P* < 0.001), and lower LV ejection fraction (*P* < 0.001) compared with Clusters 1 and 2. Cluster 2, in contrast, had notably higher LV mechanical dispersion (*P* < 0.001), higher pulmonary systolic pressures (albeit overall mild-moderate; *P* < 0.001), and a greater proportion of cases with mild-to-moderate RV systolic dysfunction (*P* < 0.001) compared with Clusters 1 and 3 (Table 2). Cluster 1 was defined by younger individuals with normal LA/LV structure and function and normal pulmonary pressures.

**FIGURE 3:**
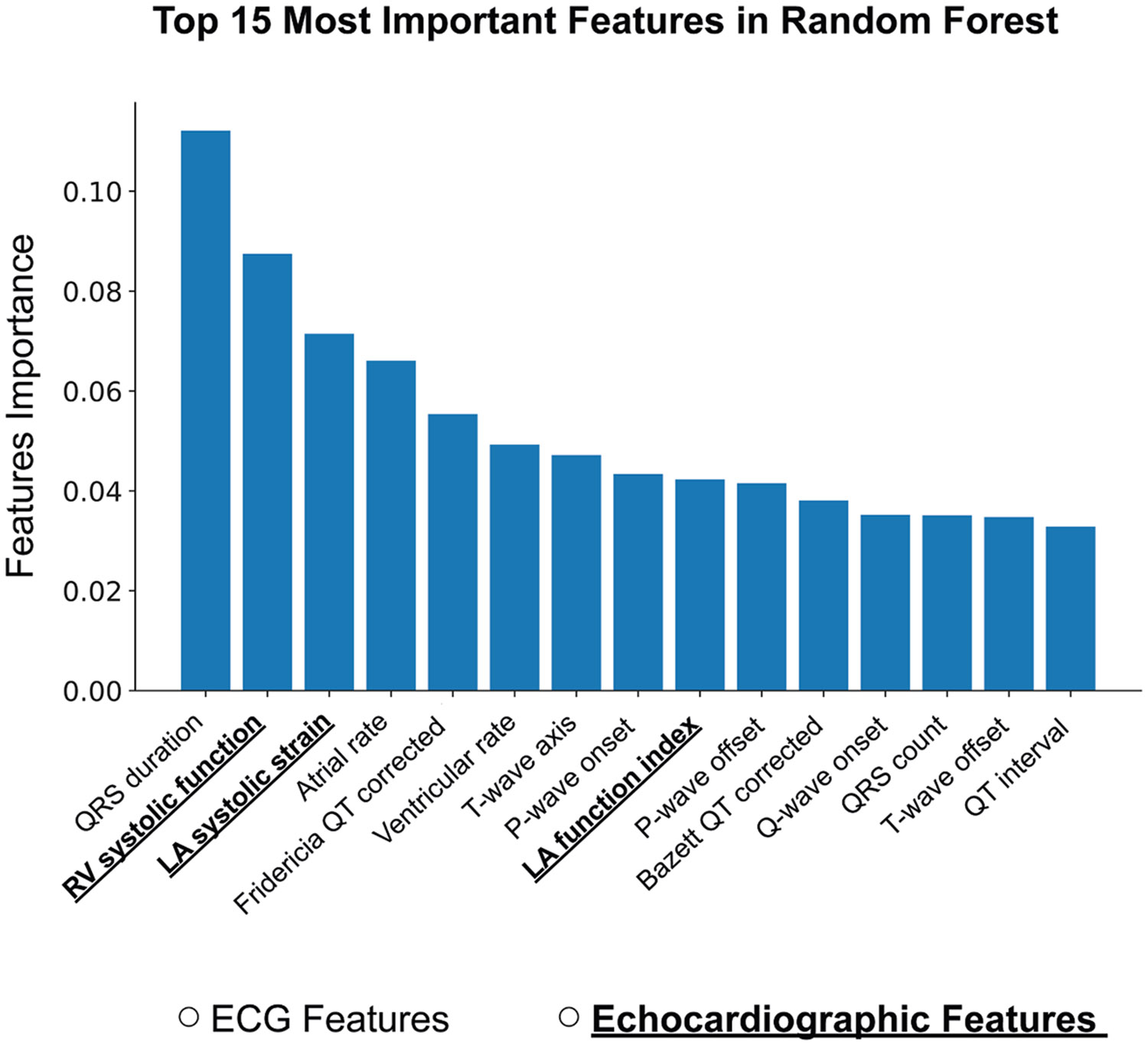
Feature Importance of HCA The top 15 of the most important features for the hierarchical clustering algorithm. Abbreviations as in Figure 1.

The most important features for clustering, as determined using the Random Forest plot, are presented in Figure 3. Among the top 15 features, we identified multiple ECG intervals related to depolarization and repolarization, alongside key echocardiographic parameters such as LA “reservoir” strain, LA function index, and RV systolic function (Figure 3). Of note, RV systolic function, atrial rate, and QRS duration were among the top 3 most important features. The features heatmap correlation is presented in Supplemental Figure 1.

### HIERARCHICAL CLUSTERING AND CLINICAL OUTCOME. Arrhythmic events.

Table 3 shows the distribution of arrhythmic events according to clusters. Overall, 77 (22%) MVPs had arrhythmic presentations, including 12 cases with SCA/ventricular fibrillation. Remaining arrhythmic cases had frequent premature ventricular contractions or sustained/non-sustained VT, alone or in combination. The distribution of the composite of arrhythmic events was significantly different between clusters: 19% in Cluster 1, 38% in Cluster 2, and 43% in Cluster 3 (*P* < 0.001; Table 3). When evaluated individually, there were significant differences between clusters with regard to both SCA and sustained/non-sustained VT (Table 3).

### Incidence of death.

**FIGURE 4:**
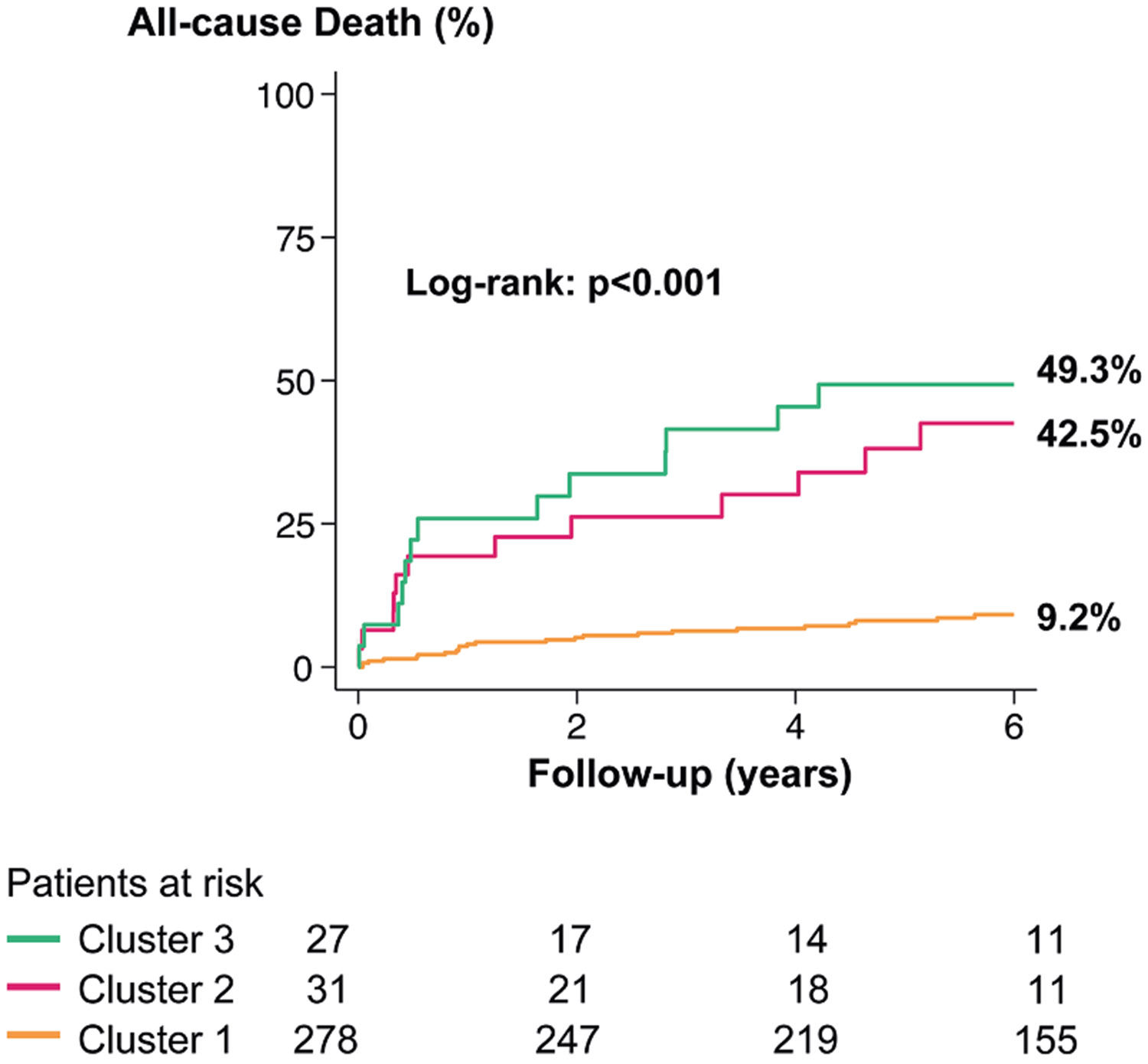
Incidence of All-Cause Death According to Clusters Kaplan-Meier curves of the cumulative incidence of all-cause death according to clusters of MVP. Numbers on the right of the curves indicate the rates of all-cause death after 6 years of follow-up. Abbreviations as in Figure 1.

During a mean follow-up of 5.4 ± 2.7 years, there were overall 65 deaths (18%), including 15 cardiac deaths, with 1 arrhythmic death in Cluster 3. There was a significant difference in the incidence of all-cause death between clusters (*P* < 0.001), with higher 6-year rates in Cluster 3 (49.3%) and Cluster 2 (42.5%) compared with Cluster 1 (9.2%) (Figure 4). Each increase in MVP cluster from Cluster 1 to 3 was associated with a 2-fold increase in the risk of all-cause death (HR: 2.55 [95% CI: 1.91-3.39]; *P* < 0.001) (Table 4). Using Cluster 1 as the reference point, there was a significant increase in the risk of all-cause death for Cluster 2 (HR: 4.99 [95% CI: 2.66-9.39]; *P* < 0.001) and Cluster 3 (HR: 5.82 [95% CI: 3.10-10.9]; *P* < 0.001) (Table 4). Similar results were observed for cardiac death, with significantly increased risk for Cluster 2 (HR: 8.22 [95% CI: 2.31-29.2]; *P* = 0.001) and Cluster 3 (HR: 11.3 [95% CI: 3.43-37.0]; *P* < 0.001). After adjustment for MV intervention (as a time-dependent variable), compared with Cluster 1, Cluster 2 (HR: 5.01 [95% CI: 2.67-9.41]; *P* < 0.001) and Cluster 3 (HR: 5.85 [95% CI: 3.11-10.9]; *P* < 0.001) remained significantly associated with an increased risk of all-cause death (Table 4).

### SENSITIVITY ANALYSES.

We performed a sensitivity clustering analysis excluding demographic variables (ie, age and sex), and including only ECG and echocardiographic parameters (Supplemental Table 1, Supplemental Figure 1). Cluster characteristics are presented in Supplemental Table 2. In this sensitivity analysis, top contributing features remained consistent compared with the original clustering analysis (Supplemental Figure 2). Arrhythmic events remained significantly more prevalent in Cluster 3 and Cluster 2 (Supplemental Table 3). Furthermore, the incidence of all-cause death remained significantly higher in Cluster 3 and Cluster 2 compared with Cluster 1 (Supplemental Figure 3, Supplemental Table 4).

To further validate our findings, we conducted additional sensitivity analyses using a nonhierarchical clustering method (Supplemental Methods). These analyses provided similar cluster risk profiles, reinforcing the results of our clustering approach (Supplemental Tables 5 to 7, Supplemental Figures 4 to 6).

## DISCUSSION

In this study of consecutive MVPs without significant MR, we applied an innovative and unbiased approach for phenotyping using hierarchical clustering. We identified 3 distinct MVP phenotypes based on ECG and echocardiography alone, delineating variations in arrhythmic events and excess mortality without the use of CMR-based fibrosis assessment (Central Illustration). Cluster 3 (ie, high-risk), notably linked to increased arrhythmic and mortality risks, exhibited advanced LA/LV remodeling and dysfunction despite lack of severe MR. Cluster 2 emerged as a medium- to high-risk phenotype, positioned between Cluster 1 (ie, low-risk) and 3, with an intermediate impact on the LV and LA, and with unique RV involvement. This approach may help identify clinically meaningful MVP phenotypes, highlighting patients who may benefit from closer, tailored clinical and imaging follow-up, even in the absence of significant MR.

### ARTIFICIAL INTELLIGENCE–BASED PHENOTYPING OF MVP.

Our findings align with recent studies that apply AI-guided algorithms to identify unique MVP phenotypes.^14,15^ However, these studies were conducted in populations predominantly consisting of patients with MVP and moderate-to-severe MR or relied on algorithms for predicting CMR-related features.^14,15^ Although these approaches are attractive, they may introduce selection bias and limit our understanding of AMVP, a condition that is often not associated with significant MR nor with a focal fibrotic response.^13,33^ In our study, we applied unsupervised ML to widely available diagnostic tools—standard 12-lead ECGs and echocardiograms—in a large set of MVPs without severe MR. Furthermore, our echocardiographic features extended beyond a traditional assessment of the MV, mitral-related morphofunctional abnormalities, or the LV to include parameters related to LA and RV size and function. We believe that combining standard 12-lead ECG and comprehensive echocardiographic data provides an unbiased and effective screening tool for identifying high-risk MVPs within a predominantly benign population, without the need for additional intensive and less cost-effective clinical tools. Moreover, unsupervised ML methods applied to comprehensive and accessible data may offer a unique opportunity to unveil novel pathways, enhancing our understanding of the complex traits related to AMVP.

### COMPLEMENTARY ROLE OF ECG AND ECHOCARDIOGRAPHY IN MVP PHENOTYPING.

The most important features in our ML algorithm included both readily accessible ECG and echocardiographic parameters. QRS and QTc duration, among the top ECG features contributing to clustering, were greater in the intermediate-risk Cluster 2. This cluster also demonstrated greater mechanical dispersion by STE. Increased mechanical dispersion has been reported in AMVP, but also in long QT syndrome as a consequence of electrical dispersion.^22,34^ These findings support the complementary role of ECG and echocardiography in enabling a holistic phenotyping of MVP, a condition characterized by an electrical in addition to a structural substrate.^10^ We have previously highlighted the prognostic role of ventricular repolarization and depolarization abnormalities using a deep learning model.^16^ However, the traditional ECG finding of inferolateral T-wave inversions on a standard 12-lead ECG is uncommon (<20%) in MVP,^16^ and complementary echocardiographic phenotyping may be needed. Understanding how electrical and structural abnormalities interplay in MVP without significant MR may provide further insights into the underlying pathologic mechanisms and inform the development of novel risk stratification approaches.

Interestingly, traditional morphofunctional abnormalities, including bileaflet involvement and MAD were not among the top contributing features of the HCA. Indeed, prior reports have underlined how isolated bileaflet MVP and MAD may not significantly affect mortality, suggesting the importance of additional risk factors at a population level.^35^ In our study, LA systolic function (ie, LA reservoir strain and function index) and RV systolic function were among the top HCA features. These findings suggest that the traditional focus on the MV may not be sufficient to capture the broader spectrum of the pathophysiology of MVP.^36^

The contribution of RV systolic function may primarily reflect age differences between clusters, as the cluster with worse RV function (ie, Cluster 2) included older individuals with higher pulmonary artery systolic pressure. Cluster 3, which included MVPs of similar age with worse LV and LA function, presented normal RV systolic function. The role of a “primary,” diffuse myopathy of LV, even in MVPs without significant MR, has been proposed by our group and others based on post-mortem, genetic, and imaging studies.^12,13,37-39^ Moreover, we have recently identified a “primary” atriopathy characterized by abnormal LA remodeling and function, particularly in individuals with bileaflet MVP, and irrespective of MR severity.^24^ Such atriopathy may result from abnormal MVP-related mechanics where traction is exerted not only the basal LV myocardium, but also on the LA wall by the prolapsing leaflets. However, this hypothesis requires further validation in future experimental studies.

### PHENOTYPING OF MVP AND CLINICAL OUTCOMES.

Previous studies using clustering approaches in MVP have demonstrated their value in predicting clinical outcomes, including heart failure, arrhythmic complications, and cardiovascular events.^14,15^ Although these studies provided information on arrhythmic risk, this was evaluated in the context of a composite outcome and not as a “pure” outcome. Moreover, the contribution to excess mortality, the most objective and universal clinical outcome, was not assessed by these studies.^14,15^ Therefore, our study is the first to identify distinct MVP phenotypes with varying arrhythmic profiles and mortality risk. Notably, we identified 1 cluster consisting of a small subset of individuals (8%), but with significantly higher risk of arrhythmic and adverse events. This high-risk cluster of MVPs was characterized by more advanced LA and LV systolic dysfunction, despite the lack of significant MR.

### CLINICAL IMPLICATIONS.

The identification of specific MVP phenotypes using an artificial intelligence– guided clustering approach provides a framework for enhanced risk stratification (Central Illustration). In particular, our findings suggest that patients in Cluster 3, characterized by abnormal ventricular repolarization/depolarization on ECG and LA/LV dysfunction on echocardiography, represent a high-risk phenotype who may benefit from more frequent clinical follow-up, as well as earlier consideration for therapeutic management. Cluster 2, an intermediate phenotype between Cluster 3 and Cluster 1, represents a less severe phenotype but still associated with an increased risk of arrhythmic events and mortality. Thus, this intermediate phenotype may also benefit from more frequent monitoring. Our integrated approach may help enhance the phenotypic characterization of MVP, enabling more personalized management for patients, especially those without severe MR who are at risk of arrhythmic complications and excess mortality. However, this approach needs to be validated using a supervised learning approach and may be further refined with the addition of other imaging parameters such as replacement myocardial fibrosis by CMR.

### STUDY LIMITATIONS.

The number of MVP cases in “high-risk” Clusters 2 and 3 were very small, reflecting the reality of clinical practice where arrhythmic events among MVP patients are rare. Hence, some comparisons may have been nonsignificant due to a small sample size within these clusters.

Although we used standard, well-validated clinical and echocardiographic features, this analysis was conducted at a single center, which may limit external validity and generalizability. Future studies will aim to externally validate the findings of our clustering approach in independent samples. Although sex was included in the clustering analysis due to its known association with AMVP, we acknowledge that using categorical variables in Ward’s clustering has methodologic limitations. However, sensitivity analysis excluding sex provided consistent results, indicating that the identified cluster phenotypes were mainly driven by ECG and echocardiographic parameters. The unsupervised learning approach, although suitable for uncovering patterns in difficult-to-characterize populations like AMVP, may have limitations when considering clinical outcome prediction and applicability. The number of clusters was chosen based on “a priori” hypothesis and separation in the dendrogram of the HCA. However, the optimal number of clusters was further quantitatively validated. Finally, a genetic predisposition to arrhythmic risk in MVP may not be excluded; however, genetic data were not available for this analysis. Further studies including genetic profiling with comprehensive imaging and ECG-based clustering approach may provide additional findings into the role of genetic factors in AMVP.

## CONCLUSIONS

An unbiased, unsupervised clustering ML-based approach identifies different phenotypes of MVP without severe MR in terms of arrhythmic presentations and excess mortality: a low-risk cluster with normal LA/LV function, an intermediate-risk cluster with abnormal RV function and elevated pulmonary pressures, and a high-risk cluster with abnormal LA/LV function (“primary” atriopathy/cardiomyopathy). The proposed algorithm, which highlights for the first time the role of a “primary” atriopathy in AMVP, holds promise for personalized management strategies in MVP. Additional studies are needed to validate our findings.

## Supplementary Material

1

## Figures and Tables

**TABLE 1:** List of Demographic, ECG, and Echocardiographic Parameters Included in HCA

Demographics (2)	ECG Parameters (18)	Echocardiographic Parameters (12)
Age	Atrial rate	MVP anatomy (bileaflet vs monoleaflet)
Sex	P-wave onset	MR grade
	P-wave offset	Mitral annular disjunction
	P-wave axis	LA volume index
	R-wave axis	LA function index
	PR interval	LA peak systolic strain (ie, “reservoir”)
	Ventricular rate	LV end-diastolic volume
	T-wave axis	LV end-systolic volume
	Q-wave onset	LV ejection fraction
	Q-wave offset	LV global longitudinal strain
	QRS duration	LV mechanical dispersion
	QRS count	RV systolic function
	T-wave offset	
	QT interval	
	Corrected QT interval Bazett formula	
	Corrected QT interval Fridericia formula	
	Global RR interval	
	QT adjusted RR interval	

(n) indicates number of parameters. Bazett formula: QTc = QT interval/√ (RR interval). Fridericia formula: QTc = QT interval/(RR interval)^1/3^.

HCA = Hierarchical Clustering Analysis; LA = left atrial; LV = left ventricular; MR = mitral regurgitation; MVP = mitral valve prolapse; RV = right ventricular.

**TABLE 2:** Characteristics of the Study Sample According to Clusters

	MVP-Cluster 1 (n = 284; 83%)	MVP-Cluster 2 (n = 31; 9%)	MVP-Cluster 3 (n = 28; 8%)	*P* Value
Clinical characteristics				
Age, y	56 ± 16	64 ± 17	65 ± 17	**0.002**
Female	158 (56)	8 (26)	10 (36)	**0.002**
Body surface area, m^2^	1.81 ± 0.23	1.89 ± 0.22	1.78 ± 0.21	0.07
Body mass index, kg/m^2^	23 ± 4	25 ± 4	23 ± 5	0.09
Non-White race	53 (20)	7 (23)	6 (21)	0.92
Hypertension	89 (31)	16 (52)	11 (39)	0.06
Diabetes mellitus	20 (7)	4 (13)	2 (7)	0.46
Smoking	93 (33)	13 (42)	14 (50)	0.13
QRS duration, ms	93 ± 14	132 ± 36	94 ± 13	**<0.001**
QT duration, ms	413 ± 33	439 ± 51	347 ± 52	**<0.001**
Corrected QT duration, ms	429 ± 28	474 ± 41	449 ± 45	**<0.001**
T-wave inversion	91 (34)	11 (39)	6 (22)	0.37
Echocardiographic characteristics				
MVP anatomy				0.31
Bileaflet	142 (51)	20 (64)	11 (39)	
Posterior	98 (35)	6 (19)	12 (43)	
Anterior	40 (14)	5 (16)	5 (18)	
MR no or mild	190 (67)	22 (71)	20 (71)	0.81
MAD inferolateral	101 (36)	8 (26)	11 (39)	0.15
LA volume index, mL/m^2^	35 ± 14	40 ± 17	42 ± 12	**0.006**
LA emptying fraction, %	54 ± 9	46 ± 18	41 ± 20	**<0.001**
LA function index	0.40 ± 0.18	0.25 ± 0.16	0.23 ± 0.16	**<0.001**
LA systolic strain, %	32 ± 8	23 ± 14	24 ± 10	**<0.001**
LV end-diastolic volume, mL/m^2^	59 ± 19	54 ± 20	56 ± 27	0.43
LV end-systolic volume, mL/m^2^	24 ± 9	26 ± 12	27 ± 15	0.26
LV mass index, g/m^2^	78 ± 20	87 ± 27	83 ± 24	0.07
LV ejection fraction, %	59 ± 6	55 ± 11	51 ± 8	**<0.001**
LV-GLS, %	−21 ± 3	−17 ± 5	−17 ± 4	**<0.001**
Mechanical dispersion, ms	60 ± 34	85 ± 42	60 ± 27	**0.005**
PASP, mm Hg	25 ± 8	44 ± 20	36 ± 16	**<0.001**
RV systolic function				**<0.001**
Normal	283 (100)	17 (55)	27 (100)	
Mildly reduced	0 (0)	9 (29)	0 (0)	
Moderately reduced	0 (0)	5 (16)	0 (0)	

Values are mean ± SD or n (%). Bold text indicates statistical significance (*P* < 0.05).

LV-GLS = LV global longitudinal strain; MAD = mitral annular disjunction; PASP = pulmonary artery systolic pressure; other abbreviations as in Table 1.

**TABLE 3:** Arrhythmic Events According to Clusters

	MVP-Cluster 1 (n = 284; 83%)	MVP-Cluster 2 (n = 31; 9%)	MVP-Cluster 3 (n = 28; 8%)	*P* Value
Composite arrhythmic events	53 (19)	12 (38)	12 (34)	**<0.001**
SCA	6 (2)	3 (10)	3 (10)	**0.01**
NSVT/VT	44 (15)	10 (35)	12 (39)	**0.001**
Frequent PVCs (≥5%)	14 (5)	1 (4)	3 (10)	0.43

Values are n (%). Bold text indicates statistical significance (*P* < 0.05). Composite of arrhythmic events included sudden cardiac arrest, NSVT/VT, or frequent PVCs.

NSVT = nonsustained ventricular tachycardia; PVCs = premature ventricular contractions; SCA = sudden cardiac arrest; VT = ventricular tachycardia; other abbreviations as in Table 1.

**TABLE 4:** Risk of All-Cause Death According to Clusters

	Unadjusted Analyses		Adjusted Analyses	
	HR (95% CI)	*P* Value	HR (95% CI)	*P* Value
Analysis including				
MVP clusters, per increase	2.55 (1.91-3.39)	<0.001	2.55 (1.91-3.40)	<0.001
Analysis including				
MVP-Cluster 1	Reference		Reference	
MVP-Cluster 2	4.99 (2.66-9.39)	<0.001	5.01 (2.67-9.40)	<0.001
MVP-Cluster 3	5.82 (3.10-10.9)	<0.001	5.85 (3.11-10.9)	<0.001

Results are HR with 95% CI. Multivariable analyses adjusted for mitral valve intervention as a time-dependent variable.

Abbreviations as in Table 1.

## PERSPECTIVES

### COMPETENCY IN MEDICAL KNOWLEDGE:

Unsupervised ML for hierarchical clustering applied to standard ECG and echocardiographic data identifies 3 distinct MVP phenotypes without severe MR, each associated with varying levels of arrhythmic risk and excess mortality.

### TRANSLATIONAL OUTLOOK:

A ML-guided approach that combines readily available and prognostic ECG and echocardiographic parameters provides additional perspectives into the underlying mechanisms linked to the risk of major complications in AMVP.

## APPENDIX

For supplemental figures and tables, please see the online version of this paper.

## ABBREVIATIONS AND ACRONYMS

AMVP: arrhythmic mitral valve prolapse
CMR: cardiac magnetic resonance
ECG: electrocardiographic
GLS: global longitudinal strain
HCA: Hierarchical Clustering Analysis
LA: left atrial/atrium
LV: left ventricular/ventricle
MAD: mitral annular disjunction
ML: machine learning
MR: mitral regurgitation
MV: mitral valve
MVP: mitral valve prolapse
RV: right ventricular
SCA: sudden cardiac arrest
SCD: sudden cardiac death
STE: speckle-tracking echocardiography
VT: ventricular tachycardia

## References

[1] Delling FN, Noseworthy PA, Adams DH, Research opportunities in the treatment of mitral valve prolapse: JACC Expert Panel. J Am Coll Cardiol. 2022;80:2331–2347. 36480975 10.1016/j.jacc.2022.09.044 PMC9981237

[2] Levine RA, Hagége AA, Judge DP, Mitral valve disease–morphology and mechanisms. Nat Rev Cardiol. 2015;12:689–710. 26483167 10.1038/nrcardio.2015.161 PMC4804623

[3] Freed LA, Benjamin EJ, Levy D, Mitral valve prolapse in the general population: the benign nature of echocardiographic features in the Framingham Heart Study. J Am Coll Cardiol. 2002;40:1298–1304. 12383578 10.1016/s0735-1097(02)02161-7

[4] Otto CM, Nishimura RA, Bonow RO, 2020 ACC/AHA Guideline for the Management of Patients With Valvular Heart Disease: Executive Summary: A Report of the American College of Cardiology/American Heart Association Joint Committee on Clinical Practice Guidelines. J Am Coll Cardiol. 2021;77:450–500. 33342587 10.1016/j.jacc.2020.11.035

[5] Vahanian A, Beyersdorf F, Praz F, 2021 ESC/EACTS Guidelines for the Management of Valvular Heart Disease. Eur Heart J. 2022;43:561–632. 34453165 10.1093/eurheartj/ehab395

[6] Nalliah CJ, Mahajan R, Elliott AD, Mitral valve prolapse and sudden cardiac death: a systematic review and meta-analysis. Heart (British Cardiac Society). 2019;105:144–151. 30242141 10.1136/heartjnl-2017-312932

[7] Basso C, Iliceto S, Thiene G, Perazzolo Marra M. Mitral valve prolapse, ventricular arrhythmias, and sudden death. Circulation. 2019;140:952–964. 31498700 10.1161/CIRCULATIONAHA.118.034075

[8] Sabbag A, Essayagh B, Barrera JDR, EHRA expert consensus statement on arrhythmic mitral valve prolapse and mitral annular disjunction complex in collaboration with the ESC Council on valvular heart disease and the European Association of Cardiovascular Imaging endorsed by the Heart Rhythm Society, by the Asia Pacific Heart Rhythm Society, and by the Latin American Heart Rhythm Society. Europace. 2022;24:1981–2003. 35951656 10.1093/europace/euac125 PMC11636573

[9] Han HC, Ha FJ, Teh AW, Mitral valve prolapse and sudden cardiac death: a systematic review. J Am Heart Assoc. 2018;7:e010584. 30486705 10.1161/JAHA.118.010584 PMC6405538

[10] Basso C, Perazzolo MM, Rizzo S, Arrhythmic mitral valve prolapse and sudden cardiac death. Circulation. 2015;132:556–566. 26160859 10.1161/CIRCULATIONAHA.115.016291

[11] Chakrabarti AK, Deshmukh A, Liang JJ, Mitral annular substrate and ventricular arrhythmias in arrhythmogenic mitral valve prolapse with mitral annular disjunction. JACC Clin Electrophysiol. 2023;9:1265–1275. 37086231 10.1016/j.jacep.2023.02.010

[12] Miller MA, Devesa A, Robson PM, Arrhythmic mitral valve prolapse with only mild or moderate mitral regurgitation: characterization of myocardial substrate. JACC Clin Electrophysiol. 2023;9:1709–1716. 37227360 10.1016/j.jacep.2023.04.011 PMC13123559

[13] Delling FN, Aung S, Vittinghoff E, Antemortem and post-mortem characteristics of lethal mitral valve prolapse among all countywide sudden deaths. JACC Clin Electrophysiol. 2021;7:1025–1034. 33640349 10.1016/j.jacep.2021.01.007 PMC8374002

[14] Huttin O, Girerd N, Jobbe-Duval A, Machine learning-based phenogrouping in MVP identifies profiles associated with myocardial fibrosis and cardiovascular events. JACC Cardiovasc Imaging. 2023;16:1271–1284. 37204382 10.1016/j.jcmg.2023.03.009

[15] Akyea RK, Figliozzi S, Lopes PM, Arrhythmic mitral valve prolapse phenotype: an unsupervised machine learning analysis using a multicenter cardiac MRI registry. Radiol Cardiothorac Imaging. 2024;6:e230247. 38900026 10.1148/ryct.230247 PMC11211946

[16] Tison GH, Abreau S, Barrios J, Identifying mitral valve prolapse at risk for arrhythmias and fibrosis from electrocardiograms using deep learning. JACC Adv. 2023;2:100446. 37936601 10.1016/j.jacadv.2023.100446 PMC10629907

[17] Freed LA, Levy D, Levine RA, Prevalence and clinical outcome of mitral-valve prolapse. N Engl J Med. 1999;341:1–7. 10387935 10.1056/NEJM199907013410101

[18] Zoghbi WA, Adams D, Bonow RO, Recommendations for non invasive evaluation of native valvular regurgitation: a report from the American Society of Echocardiography developed in collaboration with the Society for Cardiovascular Magnetic Resonance. J Am Soc Echocardiogr. 2017;30:303–371. 28314623 10.1016/j.echo.2017.01.007

[19] Lang RM, Badano LP, Mor-Avi V, Recommendations for cardiac chamber quantification by echocardiography in adults: an update from the American Society of Echocardiography and the European Association of Cardiovascular Imaging. J Am Soc Echocardiogr. 2015;28:1–39. 25559473 10.1016/j.echo.2014.10.003

[20] Bhatt A, Flink L, Lu DY, Fang Q, Bibby D, Schiller NB. Exercise physiology of the left atrium: quantity and timing of contribution to cardiac output. Am J Physiol Heart Circ Physiol. 2021;320:H575–H583. 33275524 10.1152/ajpheart.00402.2020

[21] Voigt JU, Malaescu GG, Haugaa K, Badano L. How to do LA strain. Eur Heart J Cardiovasc Imaging. 2020;21:715–717. 32548623 10.1093/ehjci/jeaa091

[22] Ermakov S, Gulhar R, Lim L, Left ventricular mechanical dispersion predicts arrhythmic risk in mitral valve prolapse. Heart (British Cardiac Society). 2019;105:1063–1069. 30755467 10.1136/heartjnl-2018-314269 PMC6658737

[23] Tastet L, Ramakrishna S, Lim LJ, Mechanical dispersion discriminates between arrhythmic and nonarrhythmic sudden death: from the POST SCD study. JACC Clin Electrophysiol. 2024;10:771–773. 38363275 10.1016/j.jacep.2024.01.002 PMC12083448

[24] Tastet L, Lim LJ, Bibby D, Primary atriopathy in mitral valve prolapse: echocardiographic evidence and clinical implications. Circ Cardiovasc Imaging. 2024;17:e016319. 38860362 10.1161/CIRCIMAGING.123.016319 PMC11187656

[25] Feeny AK, Rickard J, Trulock KM, Machine learning of 12-lead QRS waveforms to identify cardiac resynchronization therapy patients with differential outcomes. Circ Arrhythm Electrophysiol. 2020;13:e008210. 32538136 10.1161/CIRCEP.119.008210 PMC7901121

[26] Breiman L. Random forests. Machine Learning. 2001;45:5–32.

[27] Virtanen P, Gommers R, Oliphant TE, SciPy 1.0: fundamental algorithms for scientific computing in Python. Nat Methods. 2020;17:261–272. 32015543 10.1038/s41592-019-0686-2 PMC7056644

[28] Pedregosa F, Varoquaux G, Gramfort A, Scikit-learn: machine learning in Python. J Machine Learning Res. 2011;12:2825–2830.

[29] Huttin O, Pierre S, Venner C, Interactions between mitral valve and left ventricle analysed by 2D speckle tracking in patients with mitral valve prolapse: one more piece to the puzzle. Eur Heart J Cardiovasc Imaging. 2017;18:323–331. 27099279 10.1093/ehjci/jew075

[30] Haugaa KH, Smedsrud MK, Steen T, Mechanical dispersion assessed by myocardial strain in patients after myocardial infarction for risk prediction of ventricular arrhythmia. JACC Cardiovasc Imaging. 2010;3:247–256. 20223421 10.1016/j.jcmg.2009.11.012

[31] Sriram CS, Syed FF, Ferguson ME, Malignant bileaflet mitral valve prolapse syndrome in patients with otherwise idiopathic out-of-hospital cardiac arrest. J Am Coll Cardiol. 2013;62:222–230. 23563135 10.1016/j.jacc.2013.02.060

[32] Tastet L, Dixit S, Jhawar R, Interstitial fibrosis and arrhythmic mitral valve prolapse: unravelling sex-based differences. J Cardiovasc Magn Res. 2024;26:101117. 10.1016/j.jocmr.2024.101117 PMC11652916 39477155

[33] Bui AH, Roujol S, Foppa M, Diffuse myocardial fibrosis in patients with mitral valve prolapse and ventricular arrhythmia. Heart (British Cardiac Society). 2017;103:204–209. 27515954 10.1136/heartjnl-2016-309303 PMC5237392

[34] Haugaa KH, Edvardsen T, Leren TP, Gran JM, Smiseth OA, Amlie JP. Left ventricular mechanical dispersion by tissue Doppler imaging: a novel approach for identifying high-risk individuals with long QT syndrome. Eur Heart J. 2009;30:330–337. 18940888 10.1093/eurheartj/ehn466 PMC2639143

[35] Nordhues BD, Siontis KC, Scott CG, Bileaflet mitral valve prolapse and risk of ventricular dysrhythmias and death. J Cardiovasc Electrophysiol. 2016;27:463–468. 26749260 10.1111/jce.12914

[36] Delling FN, Vasan RS. Epidemiology and pathophysiology of mitral valve prolapse: new insights into disease progression, genetics, and molecular basis. Circulation. 2014;129:2158–2170. 24867995 10.1161/CIRCULATIONAHA.113.006702 PMC4052751

[37] Han HC, Parsons SA, Teh AW, Characteristic histopathological findings and cardiac arrest rhythm in isolated mitral valve prolapse and sudden cardiac death. J Am Heart Assoc. 2020;9:e015587. 32233752 10.1161/JAHA.119.015587 PMC7428599

[38] Roselli C, Yu M, Nauffal V, Genome-wide association study reveals novel genetic loci: a new polygenic risk score for mitral valve prolapse. Eur Heart J. 2022;43:1668–1680. 35245370 10.1093/eurheartj/ehac049 PMC9649914

[39] Mahajan AM, Itan Y, Cerrone M, Sudden cardiac arrest in a patient with mitral valve prolapse and LMNA and SCN5A mutations. JACC Case Rep. 2021;3:242–246. 34317510 10.1016/j.jaccas.2020.11.046 PMC8310969

