# Supplementary material for: Phenotyping of Mitral Valve Prolapse Without Severe Mitral Regurgitation Using Electrocardiographic and Echocardiographic Data
Source: JACC Clin Electrophysiol. Author manuscript; Available in PMC 2026 Jun 22. (PMC13284840; doi:10.1016/j.jacep.2025.06.001)
Supplement: 1 [file NIHMS2177649-supplement-1.docx]

**SUPPLEMENTAL MATERIAL:** Phenotyping of Mitral Valve Prolapse Without Severe Mitral Regurgitation Using Electrocardiographic and Echocardiographic Data

*Lionel Tastet, Ph.D.^1^; *Minhaj U. Ansari, MS.c. ^1^; Joshua P. Barrios, Ph.D.^1^; Luca Cristin, M.D.^1^; Rohit Jhawar, B.A.^1^; Amy Rich, B.A.^1^; Dwight Bibby, R.D.C.S^1^; Qizhi Fang, M.D.^1^; Farzin Arya, MD^1^; †Geoffrey H. Tison, M.D., M.P.H.^1^; †Francesca N. Delling, M.D., M.P.H.^1^

^1^Department of Medicine (Cardiovascular Division), University of California, San Francisco, California, USA.

*L.T. and M.U.A. are co-first authors

†F.N.D. and G.H.T. are co-senior authors

***Supplemental Methods (Sensitivity Analyses)***

Sensitivity analyses excluding demographic features (age and sex) from hierarchical clustering analysis were performed to evaluate cluster stability.

***Hierarchical Clustering Analysis***

Hierarchical Cluster Analysis (HCA) is an unsupervised machine learning technique used to group similar observations into hierarchical clusters based on a similarity criterion. It starts by treating each data point as its own cluster and iteratively merges the most similar clusters until all data points are merged into a single cluster.

***Non-Hierarchical Clustering Analysis***

We performed an additional sensitivity analysis using a non-hierarchical clustering method called K-Means and excluding demographic variables (i.e., age and sex). K-Means is an unsupervised clustering algorithm that groups data into K clusters by iteratively assigning points to the nearest cluster centroid and updating centroids based on the mean of assigned points. This process continues until the centroids stabilize or a stopping criterion is met. It aims to minimize intra-cluster variance.

***Statistical Analysis***

The estimates of cumulative incidence of all-cause death according to clusters were calculated using the Kaplan-Meier method and compared using the log-rank test. The association between clusters and the risk of all-cause death was determined using Cox proportional hazards models. The multivariable model was adjusted for age and MV intervention (as a time-dependent variable). The selection of the variables was based on their clinical relevance and/or because they were considered as confounding factors. Results were presented as hazards ratio (HR) with 95% CIs.

| **Supplemental Table 1:** List of ECG and Echocardiographic Parameters included in the Hierarchical Clustering Analysis Excluding Age and Sex | |
| --- | --- |
| **Variables** |  |
| ***ECG Parameters (17)*** | ***Echocardiographic Parameters (11)*** |
| Atrial rate | MVP anatomy |
| P wave onset | Mitral regurgitation grade |
| P wave offset | LA volume index |
| P wave axis | LA function index |
| R wave axis | LA peak systolic strain (i.e. “reservoir”) |
| PR interval | LV end-diastolic volume |
| Ventricular rate | LV end-systolic volume |
| T wave axis | LV ejection fraction |
| Q wave onset | LV global longitudinal strain |
| Q wave offset | LV mechanical dispersion |
| QRS duration | RV systolic function |
| QRS count |  |
| T wave offset |  |
| QT interval |  |
| Corrected QT interval Bazett formula |  |
| Corrected QT interval Fridericia formula |  |
| Global RR interval |  |
| Bazett formula: QTc = QT interval / √ (RR interval). Fridericia formula: QTc = QT interval / (RR interval)^1/3^.  LA = left atrial; LV = left ventricular; MVP = mitral valve prolapse; RV = right ventricular. (n) indicates number of parameters. | |

***Supplemental Results (Sensitivity Analyses)***

| **Supplemental Table 2:** Characteristics of the Study Sample According to Clusters (Ward’s Method excluding age and sex) | | | | |
| --- | --- | --- | --- | --- |
|  | **MVP-Cluster 1**  **(n = 217; 63%)** | **MVP-Cluster 2**  **(n = 103; 30%)** | **MVP-Cluster 3**  **(n = 23; 7%)** | **p Value** |
| ***Clinical characteristics*** |  |  |  |  |
| Age, years | 56 ± 16 | 60 ± 15 | 64 ± 18 | **0.01** |
| Female, n (%) | 116 (53) | 51 (50) | 9 (39) | 0.39 |
| Body surface area, m² | 1.83 ± 0.22 | 1.78 ± 0.23 | 1.84 ± 0.22 | 0.14 |
| Body mass index, kg/m² | 24 ± 4 | 23 ± 4 | 25 ± 4 | 0.26 |
| Non-White race, n (%) | 34 (17) | 29 (29) | 3 (13) | **0.03** |
| Hypertension, n (%) | 60 (28) | 46 (45) | 10 (43) | **0.007** |
| Diabetes mellitus, n (%) | 11 (5) | 11 (11) | 4 (17) | **0.04** |
| Smoking, n (%) | 68 (31) | 42 (41) | 10 (43) | 0.17 |
| QRS duration, ms | 93 ± 12 | 93 ± 14 | 152 ± 26 | **<0.001** |
| QT duration, ms | 420 ± 30 | 377 ± 40 | 463 ± 44 | **<0.001** |
| Corrected QT duration, ms | 422 ± 25 | 447 ± 34 | 493 ± 37 | **<0.001** |
| T wave inversion, n (%) | 61 (29) | 41 (41) | 6 (33) | 0.06 |
| ***Echocardiographic characteristics*** |  |  |  |  |
| MVP anatomy |  |  |  | 0.61 |
| Bileaflet, n (%) | 110 (51) | 53 (53) | 10 (43) |  |
| Posterior, n (%) | 75 (35) | 34 (34) | 7 (30) |  |
| Anterior, n (%) | 31 (14) | 13 (13) | 6 (26) |  |
| MR no or mild, n (%) | 131 (60) | 87 (84) | 14 (61) | **<0.001** |
| MAD inferolateral, n (%) | 101 (36) | 8 (26) | 11 (39) | 0.15 |
| LA volume index, ml/m^2^ | 35 ± 14 | 38 ± 14 | 38 ± 18 | 0.18 |
| LA emptying fraction, % | 54 ± 10 | 50 ± 13 | 47 ± 18 | **0.01** |
| LA function index | 0.40 ± 0.18 | 0.33 ± 0.17 | 0.33 ± 0.27 | **0.01** |
| LA systolic strain, % | 32 ± 8 | 28 ± 9 | 25 ± 14 | **<0.001** |
| LV end-diastolic volume, ml/m^2^ | 60 ± 19 | 56 ± 20 | 54 ± 19 | 0.17 |
| LV end-systolic volume, ml/m^2^ | 25 ± 9 | 25 ± 12 | 25 ± 11 | 0.96 |
| LV mass index, g/m^2^ | 78 ± 19 | 80 ± 24 | 90 ± 28 | 0.06 |
| LV ejection fraction, % | 59 ± 6 | 56 ± 8 | 55 ± 10 | **0.001** |
| LV-GLS, % | -21 ± 3 | -19 ± 4 | -17 ± 4 | **<0.001** |
| Mechanical dispersion, ms | 60 ± 36 | 59 ± 25 | 88 ± 43 | **0.002** |
| PASP, mmHg | 25 ± 8 | 28 ± 10 | 45 ± 22 | **<0.001** |
| RV systolic function |  |  |  | **<0.001** |
| Normal, n (%) | 211 (98) | 100 (98) | 16 (70) |  |
| Midly reduced, n (%) | 5 (2) | 2 (2) | 2 (9) |  |
| Moderately reduced, n (%) | 0 (0) | 0 (0) | 5 (21) |  |
| Values are mean ± SD  LV-GLS = LV global longitudinal strain; MAD = mitral annular disjunction; MR = mitral regurgitation; PASP = pulmonary artery systolic pressure; other abbreviations as in **Supplemental** **Table 1**. Bold text indicates statistical significance (p<0.05). | | | | |

| **Supplemental Table 3:** Arrhythmic Events According to Clusters (Ward’s Method excluding Age and Sex) | | | | |
| --- | --- | --- | --- | --- |
|  | **MVP-Cluster 1**  **(n = 217; 63%)** | **MVP-Cluster 2**  **(n = 103; 30%)** | **MVP-Cluster 3**  **(n = 23; 7%)** | **p Value** |
| Composite arrhythmic events, n (%) | 39 (17) | 30 (29) | 8 (35) | **0.02** |
| Sudden cardiac arrest, n (%) | 5 (2) | 7 (7) | 0 (0) | 0.10 |
| NSVT/VT, n (%) | 31 (14) | 27 (26) | 8 (35) | **0.006** |
| Frequent PVCs (≥5%), n (%) | 12 (5) | 4 (3) | 2 (9) | 0.48 |
| Composite of arrhythmic events included sudden cardiac arrest, non-sustained ventricular tachycardia (NSVT)/ventricular tachycardia (VT), or frequent premature ventricular contractions (PVCs). Other abbreviations as in **Supplemental** **Table 1**. Bold text indicates statistical significance (p<0.05). | | | | |

| **Supplemental Table 4:** Risk of All-Cause Death According to Clusters (Ward’s Method excluding Age and Sex) | | | | | |
| --- | --- | --- | --- | --- | --- |
|  | **Unadjusted Analyses** | |  | **Adjusted Analyses** | |
| **Variables** | **HR (95% CI)** | **p Value** |  | **HR (95% CI)** | **p Value** |
| ***Analysis including*** |  |  |  |  |  |
| MVP clusters, per increase | 2.36 (1.68 - 3.32) | <0.001 |  | 2.06 (1.46 - 2.91) | <0.001 |
| ***Analysis including*** |  |  |  |  |  |
| MVP-Cluster 1 | Reference |  |  | Reference |  |
| MVP-Cluster 2 | 2.73 (1.58 - 4.74) | <0.001 |  | 2.56 (1.46 - 4.49) | 0.001 |
| MVP-Cluster 3 | 5.21 (2.49 - 10.9) | <0.001 |  | 3.85 (1.81 - 8.21) | <0.001 |
| Results are hazard ratio (HR) with 95% confidence interval (CI). Multivariable analyses adjusted for age and mitral valve intervention as a time-dependent variable.  Abbreviations as in **Supplemental** **Table 1**. | | | | | |

| **Supplemental Table 5:** Characteristics of the Study Sample According to Clusters (Non-Hierarchical Analysis) | | | | |
| --- | --- | --- | --- | --- |
|  | **MVP-Cluster 1**  **(n = 212; 62%)** | **MVP-Cluster 2**  **(n = 95; 28%)** | **MVP-Cluster 3**  **(n = 36; 10%)** | **p Value** |
| ***Clinical characteristics*** |  |  |  |  |
| Age, years | 56 ± 15 | 56 ± 16 | 66 ± 15 | **0.001** |
| Female, n (%) | 115 (54) | 48 (51) | 13 (36) | 0.13 |
| Body surface area, m² | 1.83 ± 0.22 | 1.76 ± 0.22 | 1.87 ± 0.22 | **0.02** |
| Body mass index, kg/m² | 23 ± 4 | 23 ± 4 | 24 ± 4 | 0.46 |
| Non-White race, n (%) | 36 (18) | 26 (28) | 4 (11) | **0.04** |
| Hypertension, n (%) | 57 (27) | 41 (43) | 18 (50) | **0.002** |
| Diabetes mellitus, n (%) | 13 (6) | 10 (11) | 3 (9) | 0.37 |
| Smoking, n (%) | 67 (32) | 36 (38) | 17 (47) | 0.15 |
| QRS duration, ms | 94 ± 14 | 89 ± 11 | 131 ± 33 | **<0.001** |
| QT duration, ms | 424 ± 28 | 365 ± 33 | 444 ± 43 | **<0.001** |
| Corrected QT duration, ms | 424 ± 27 | 441 ± 33 | 479 ± 37 | **<0.001** |
| T wave inversion, n (%) | 69 (34) | 30 (33) | 9 (28) | 0.77 |
| ***Echocardiographic characteristics*** |  |  |  |  |
| MVP anatomy |  |  |  | 0.95 |
| Bileaflet, n (%) | 107 (51) | 46 (50) | 20 (56) |  |
| Posterior, n (%) | 73 (35) | 31 (34) | 12 (33) |  |
| Anterior, n (%) | 31 (15) | 15 (16) | 4 (11) |  |
| MR no or mild, n (%) | 135 (64) | 70 (74) | 27 (75) | 0.14 |
| MAD inferolateral, n (%) | 73 (34) | 41 (43) | 6 (17) | **0.02** |
| LA volume index, ml/m^2^ | 35 ± 14 | 36 ± 13 | 43 ± 16 | **0.005** |
| LA emptying fraction, % | 54 ± 9 | 52 ± 12 | 41 ± 18 | **<0.001** |
| LA function index | 0.42 ± 0.19 | 0.35 ± 0.17 | 0.22 ± 0.12 | **<0.001** |
| LA systolic strain, % | 32 ± 8 | 30 ± 11 | 21 ± 9 | **<0.001** |
| LV end-diastolic volume, ml/m^2^ | 59 ± 19 | 58 ± 18 | 56 ± 24 | 0.74 |
| LV end-systolic volume, ml/m^2^ | 24 ± 9 | 26 ± 9 | 27 ± 15 | 0.10 |
| LV mass index, g/m^2^ | 79 ± 20 | 77 ± 28 | 91 ± 28 | **0.004** |
| LV ejection fraction, % | 60 ± 6 | 56 ± 7 | 53 ± 12 | **<0.001** |
| LV-GLS, % | -21 ± 3 | -19 ± 3 | -17 ± 5 | **<0.001** |
| Mechanical dispersion, ms | 61 ± 35 | 54 ± 25 | 93 ± 44 | **<0.001** |
| PASP, mmHg | 25 ± 8 | 27 ± 10 | 43 ± 19 | **<0.001** |
| RV systolic function |  |  |  | **<0.001** |
| Normal, n (%) | 209 (99) | 91 (97) | 27 (75) |  |
| Midly reduced, n (%) | 2 (1) | 3 (3) | 4 (11) |  |
| Moderately reduced, n (%) | 0 (0) | 0 (0) | 5 (14) |  |
| Values are mean ± SD  LV-GLS = LV global longitudinal strain; MAD = mitral annular disjunction; MR = mitral regurgitation; PASP = pulmonary artery systolic pressure; other abbreviations as in **Supplemental** **Table 1**. Bold text indicates statistical significance (p<0.05). | | | | |

| **Supplemental Table 6:** Arrhythmic Events According to Clusters (Non-Hierarchical Analysis) | | | | |
| --- | --- | --- | --- | --- |
|  | **MVP-Cluster 1**  **(n = 212; 62%)** | **MVP-Cluster 2**  **(n = 95; 28%)** | **MVP-Cluster 3**  **(n = 36; 10%)** | **p Value** |
| Composite arrhythmic events, n (%) | 42 (19) | 20 (21) | 15 (42) | **0.01** |
| Sudden cardiac arrest, n (%) | 6 (3) | 4 (4) | 2 (6) | 0.44 |
| NSVT/VT, n (%) | 34 (16) | 17 (18) | 15 (42) | **0.001** |
| Frequent PVCs (≥5%), n (%) | 12 (6) | 3 (3) | 3 (8) | 0.40 |
| Composite of arrhythmic events included sudden cardiac arrest, non-sustained ventricular tachycardia (NSVT)/ventricular tachycardia (VT), or frequent premature ventricular contractions (PVCs). Other abbreviations as in **Supplemental** **Table 1**. Bold text indicates statistical significance (p<0.05). | | | | |

| **Supplemental Table 7:** Risk of All-Cause Death According to Clusters (Non-Hierarchical Analysis) | | | | | |
| --- | --- | --- | --- | --- | --- |
|  | **Unadjusted Analyses** | |  | **Adjusted Analyses** | |
| **Variables** | **HR (95% CI)** | **p Value** |  | **HR (95% CI)** | **p Value** |
| ***Analysis including*** |  |  |  |  |  |
| MVP clusters, per increase | 2.18 (1.59 - 2.98) | <0.001 |  | 1.71 (1.24 - 2.36) | <0.001 |
| ***Analysis including*** |  |  |  |  |  |
| MVP-Cluster 1 | Reference |  |  | Reference |  |
| MVP-Cluster 2 | 2.44 (1.37 - 4.37) | 0.002 |  | 2.40 (1.33 - 4.33) | 0.003 |
| MVP-Cluster 3 | 4.64 (2.44 - 8.87) | <0.001 |  | 2.71 (1.37 - 5.38) | 0.004 |
| Results are hazard ratio (HR) with 95% confidence interval (CI). Multivariable analyses adjusted for age, sex, and mitral valve intervention as a time-dependent variable.  Abbreviations as in **Supplemental** **Table 1**. | | | | | |

**SUPPLEMENTAL FIGURE 1**


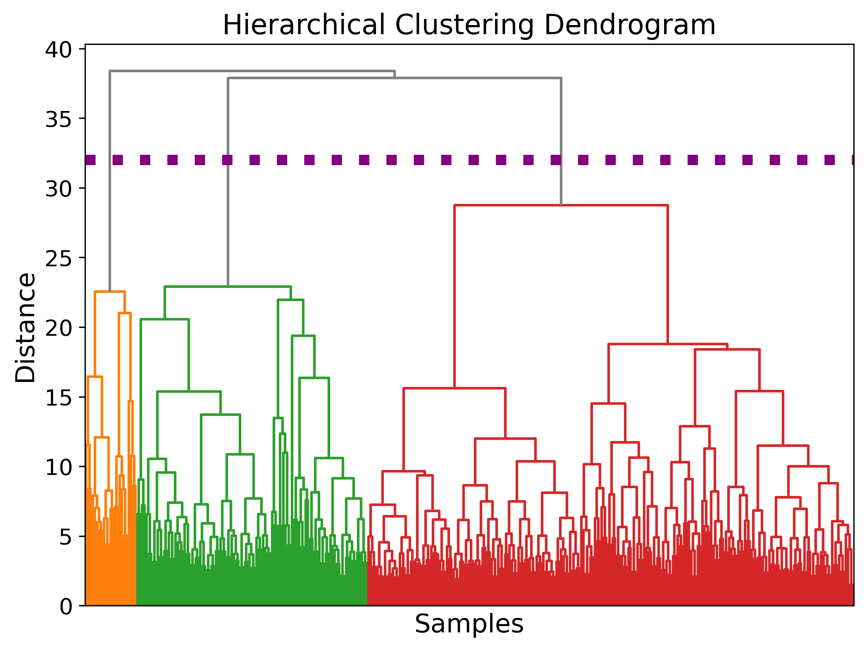


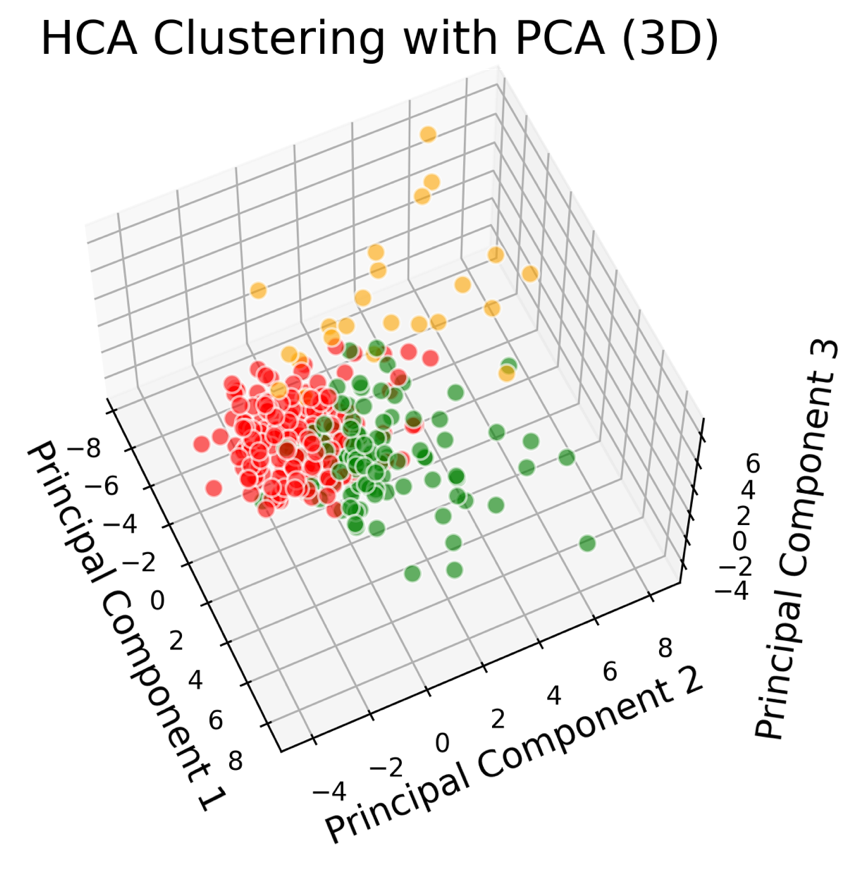


***Caption***: Visualization of hierarchical clustering analysis (**top**) and principal component analysis (PCA) (**bottom**), when excluding demographic data (age and sex).

**SUPPLEMENTAL FIGURE 2**


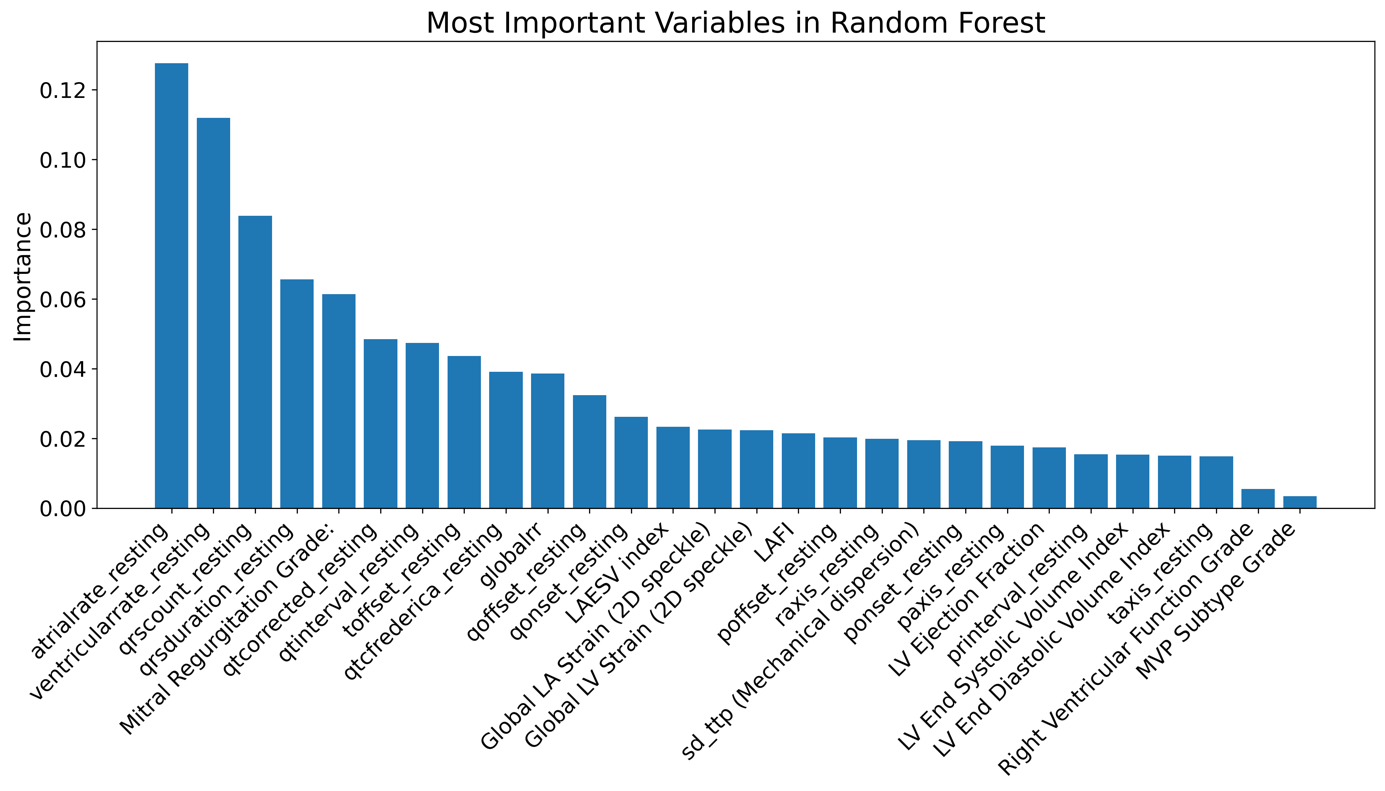


***Caption***: The most important features for the hierarchical clustering algorithm when excluding demographic data.

**SUPPLEMENTAL FIGURE 3**


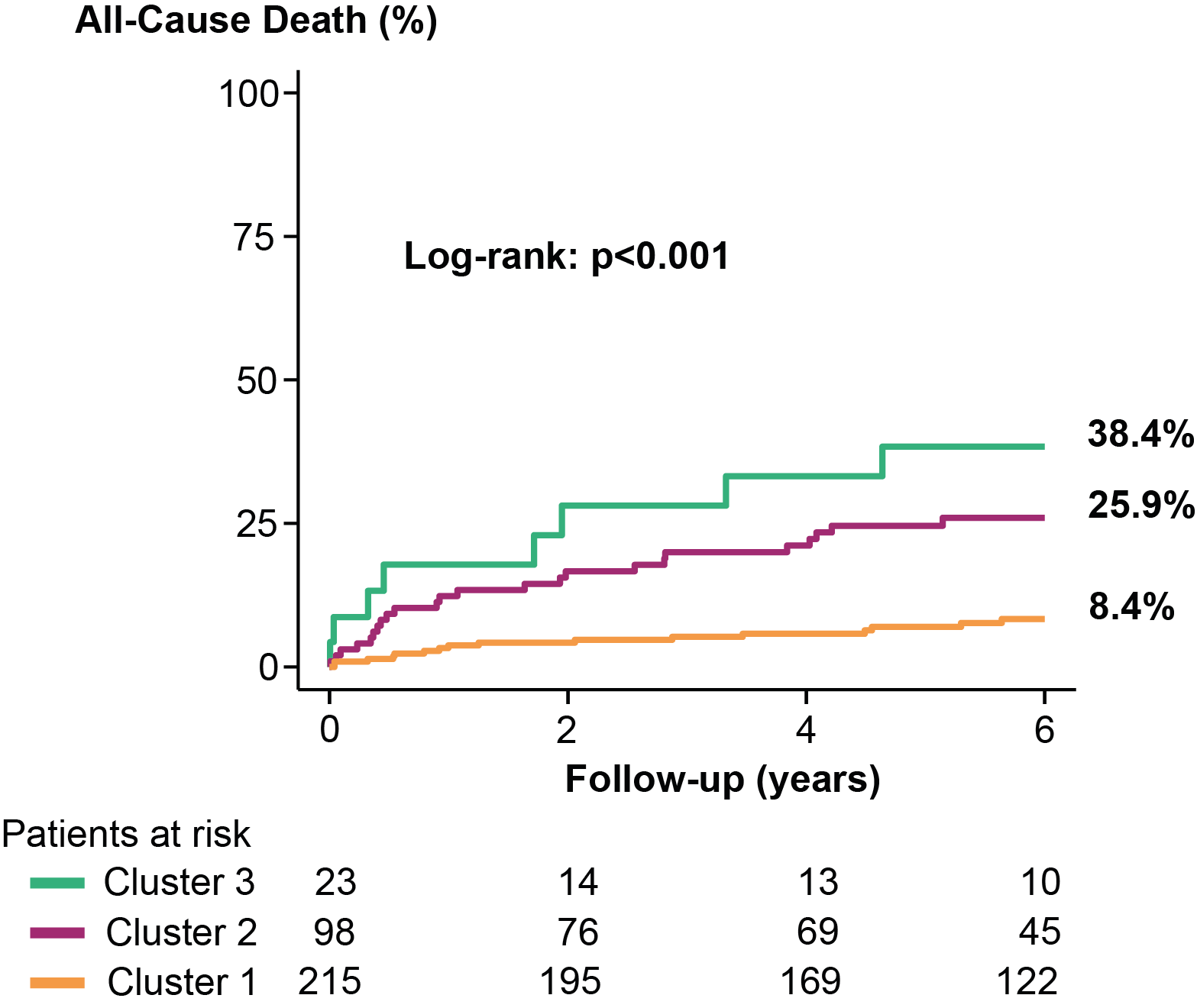


***Caption***: Kaplan-Meier curves of the cumulative incidence of all-cause death according to clusters after hierarchical clustering analysis excluding demographic data. Numbers on the right of the curves indicate the rates of all-cause death after 6 years of follow-up.

**SUPPLEMENTAL FIGURE 4**


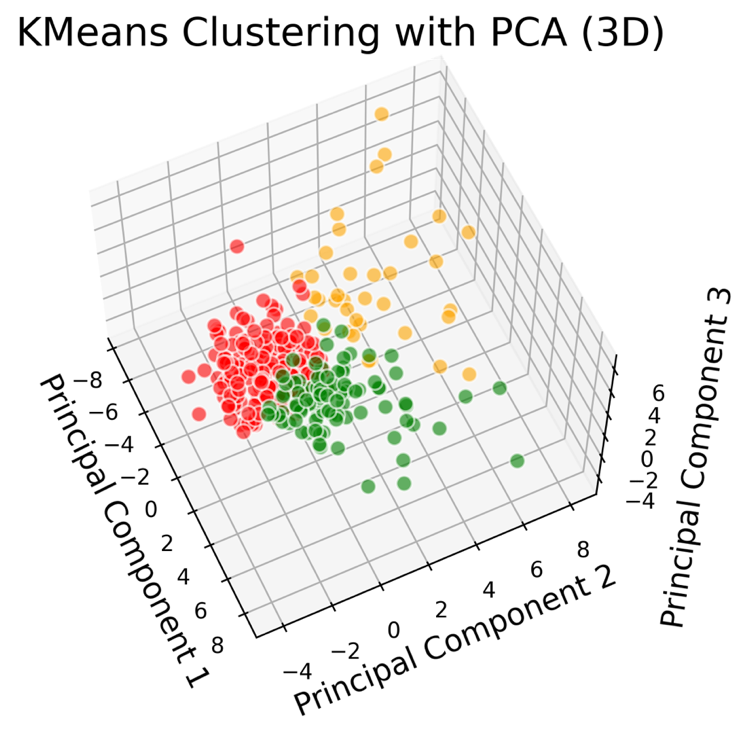


***Caption***: Principal component analysis following non-hierarchical clustering (i.e., Kmeans clustering) excluding demographic data.

**SUPPLEMENTAL FIGURE 5**


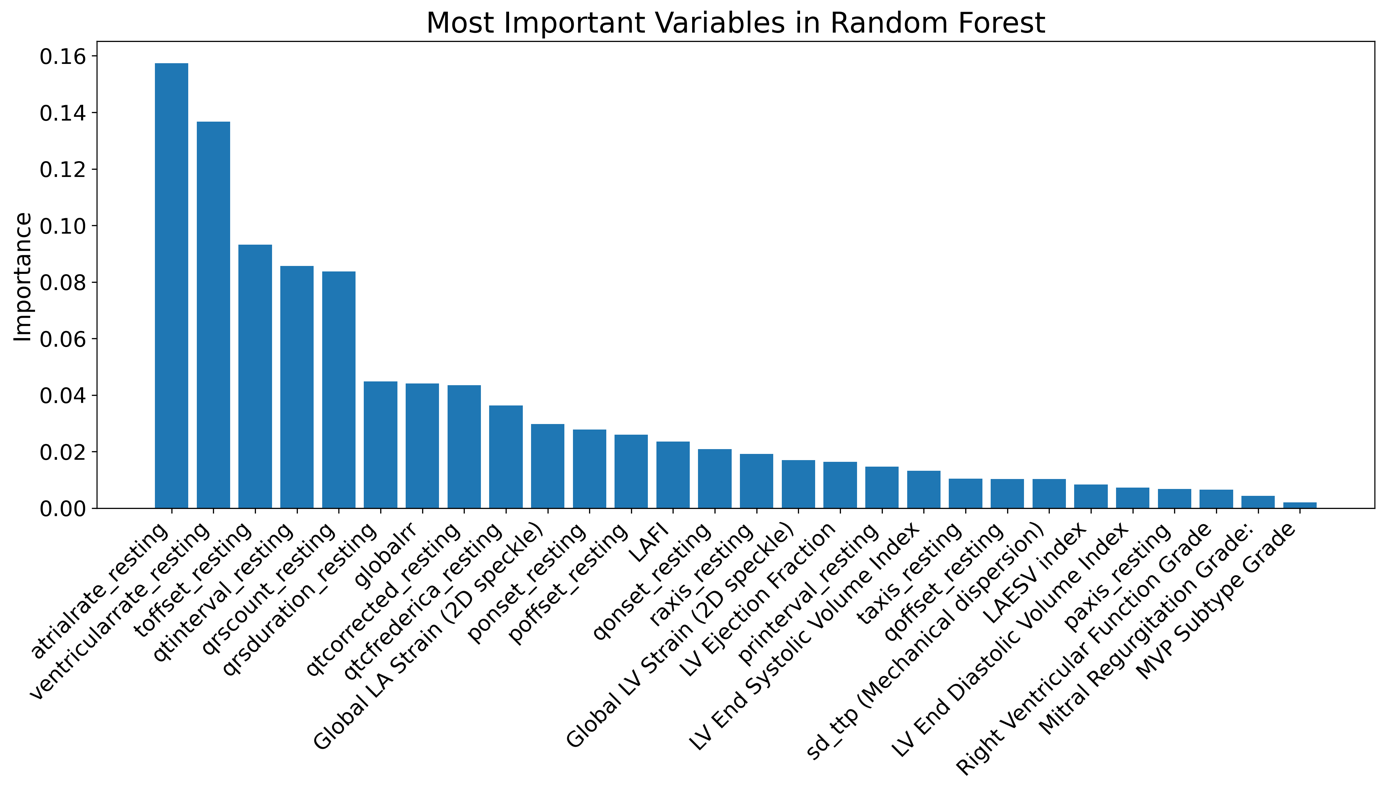


***Caption***: The most important features for the non-hierarchical clustering analysis (excluding demographic data).

**SUPPLEMENTAL FIGURE 6**


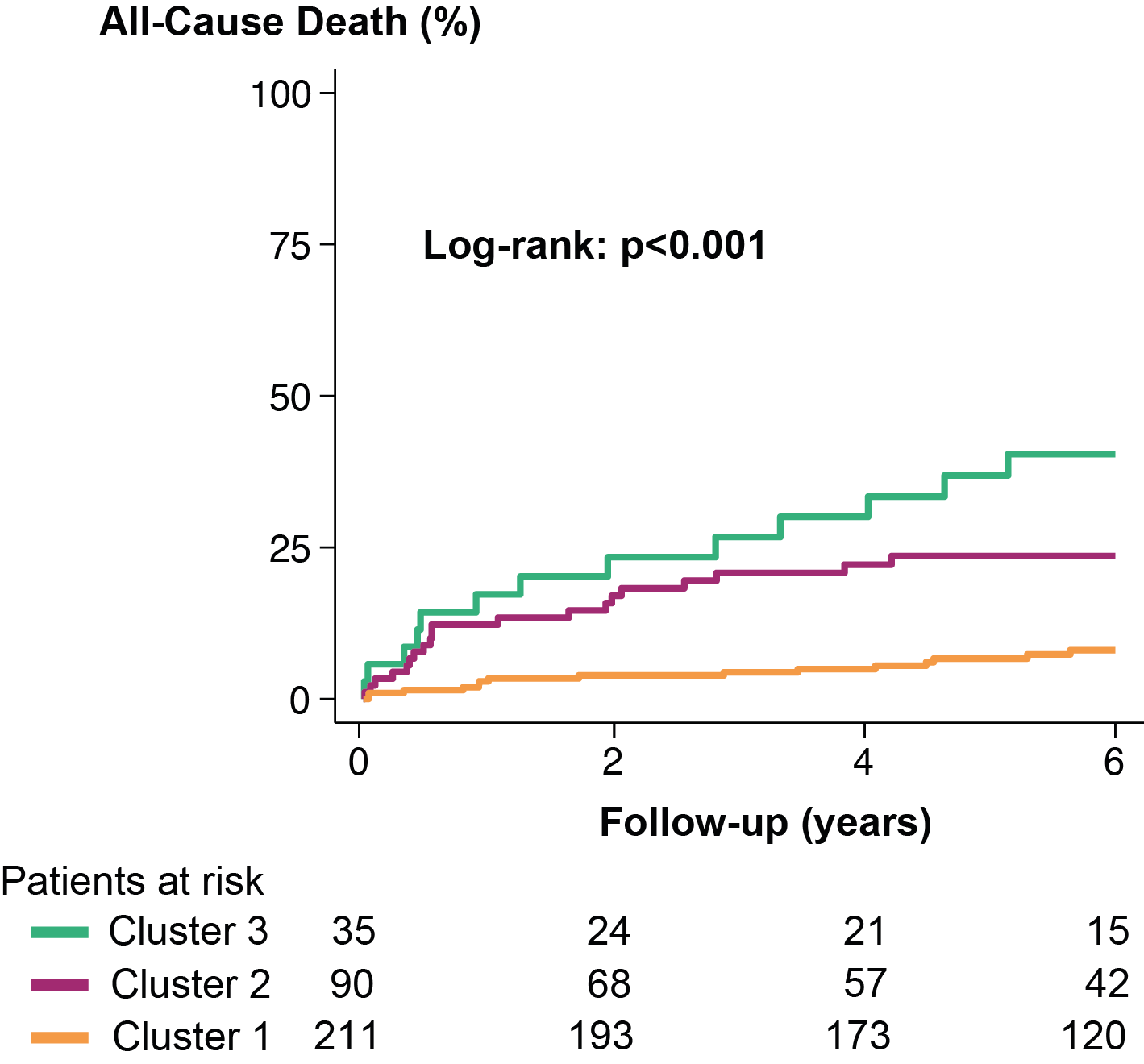


***Caption***: Kaplan-Meier curves of the cumulative incidence of all-cause death according to clusters after non-hierarchical clustering analysis (excluding demographic data). Numbers on the right of the curves indicate the rates of all-cause death after 6 years of follow-up.
